# Numerical Study on the Potential of Cavitation Damage in a Lead–Bismuth Eutectic Spallation Target

**DOI:** 10.3390/ma12040681

**Published:** 2019-02-25

**Authors:** Tao Wan, Takashi Naoe, Hiroyuki Kogawa, Masatoshi Futakawa, Hironari Obayashi, Toshinobu Sasa

**Affiliations:** J-PARC Center, Japan Atomic Energy Agency, 2-4 Shirakata, Tokai-mura, Ibaraki 319-1195, Japan; naoe.takashi@jaea.go.jp (T.N.); kogawa.hiroyuki@jaea.go.jp (H.K.); futakawa.masatoshi@jaea.go.jp (M.F.); obayashi.hironari@jaea.go.jp (H.O.); sasa.toshinobu@jaea.go.jp (T.S.)

**Keywords:** cavitation, bubble dynamics, ADS, lead–bismuth, spallation target, J-PARC

## Abstract

To perform basic Research and Development for future Accelerator-driven Systems (ADSs), Japan Proton Accelerator Research Complex (J-PARC) will construct an ADS target test facility. A Lead–Bismuth Eutectic (LBE) spallation target will be installed in the target test facility and bombarded by pulsed proton beams (250 kW, 400 MeV, 25 Hz, and 0.5 ms pulse duration). To realize the LBE spallation target, cavitation damage due to pressure changes in the liquid metal should be determined, preliminarily, because such damage is considered to be very critical, from the viewpoint of target safety and lifetime. In this study, cavitation damage due to pressure waves caused by pulsed proton beam injection and turbulent liquid metal flow, were studied, numerically, from the viewpoint of single cavitation bubble dynamics. Specifically, the threshold of cavitation and effects of flow speed fluctuation on cavitation bubble dynamics, in an orifice structure, were investigated in the present work. The results showed that the LBE spallation target did not undergo cavitation damage, under normal nominal operation conditions, mainly because of the long pulse duration of the pulsed proton beam and the low liquid metal flow velocity. Nevertheless, the possibility of cavitation damage in the orifice structure, under certain extreme transient LBE flow conditions, cannot be neglected.

## 1. Introduction

Heavy Liquid Metals (HLM) are usually employed as spallation materials and coolants in spallation targets, which are key components of neutron sources and Accelerator-Driven Systems (ADSs) [1,2]. In such systems, high-power pulsed proton beams are injected into the HLM to initiate the spallation reaction, to produce neutrons exhibiting various spectra for frontier scientific and industrial applications. However, the temperature of HLM rises very rapidly owing to cyclic and intense deposition of the energy of pulsed proton beams in liquid metal, which can lead to severe problems. The HLM volume expands sharply and high positive pressure is generated, as a result. The propagation of positive pressure toward the surrounding liquid creates a negative pressure zone, which might drive expansion of the cavitation bubble core. The collapse of a cavitation bubble close to the HLM container wall, after reaching its maximum radius, causes cavitation damage on the wall. Moreover, it is well-known that, in addition to the pressure waves, turbulent HLM flow can create a negative pressure in liquid. Especially, when the liquid flow passes through an orifice-like structure, for example, the cross-section of the liquid flow path expands or shrinks suddenly, the liquid flow detaches at the downstream position of the orifice; subsequently, a second flow is driven toward the inverse flow direction of the main flow; and finally, a circular flow is formed. As a result, the pressure of the liquid drops, owing to the formation of this flow vortex, which eventually leads to cavitation damage on the walls of liquid container.

It has been reported that the pulsed spallation neutron targets at the J-PARC (Japan Proton Accelerator Research Complex), and at Oak Ridge National Laboratory have undergone severe cavitation damage [3,4]. The cavitation damage has severely eroded the spallation target vessels and was considered to be a significant factor leading to shortening of the target lifetime and eventual lifetime. In the two facilities, mercury is employed as the spallation/coolant material and is bombarded by pulsed proton beams of different profiles.

As for solving the basic technical issues related to future ADSs, for example, Lead–Bismuth Eutectic (LBE) flow behavior and structural material performance under high temperature, intense irradiation, and LBE flow conditions, construction of the ADS target test facility will be conducted within the framework of J-PARC [5]. In the target test facility, a LBE spallation target will be installed. The primary candidate material for constructing the target vessel is type 316 austenitic stainless steel (SS), as a database of its mechanical properties of post-irradiation examination under proton/neutron has already been created [6]. Pulsed proton beams (250 kW, 400 MeV, 25 Hz, 0.5 ms in pulse duration) will be injected into the spallation target to induce spallation reactions for various applications [7]. Moreover, it is very important to clarify the cavitation issue at the preliminary stages, during the design and construction of the LBE spallation target for the target test facility.

In the present study, by means of a numerical simulation, we investigated the possibility of occurrence of cavitation damage to the LBE spallation target, due to pressure waves and turbulent LBE flow. The initialization threshold of cavitation damage was studied by considering single bubble dynamics in a pressure oscillation field. The effects of flow fluctuation on cavitation bubble dynamics in an orifice-like structure are discussed as well.

## 2. Configuration of LBE Spallation Target Head

**Figure 1:**
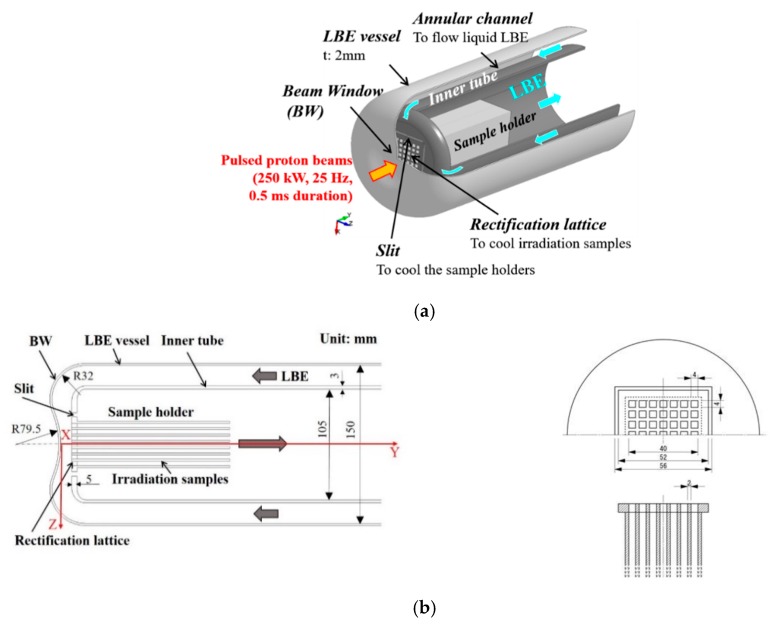
Schematic drawing and dimensions of design of the Lead–Bismuth Eutectic (LBE) spallation target head: (**a**) Schematic drawing of LBE spallation target head; and (**b**) dimensions of the LBE spallation target head.

Figure 1 shows a schematic drawing and dimensions of the designed LBE spallation target head. The length of the target head is 600 mm. The target has a Beam Window (BW). The BW is to be made of type 316 SS with a uniform thickness of 2 mm, during the first-phase operation of the LBE spallation target. A coaxially arranged annular flow channel is provided between the LBE vessel and the inner tube to follow the liquid LBE to the BW. The inner diameter of the LBE vessel and the inner tube are 150 mm and 105 mm, respectively. A concave surface with a radius of 32 mm is provided on the upper part of the BW, to facilitate return flow of the LBE, while the center part of the BW is made of a convex surface that curves in the opposite direction, with a radius of 79.5 mm. Eight plate-type irradiation samples are located in front of the inner tube, with intervals of 4 mm in the Z-direction, and the sample holder is set to support the irradiation samples. The size of each irradiation sample is 40 × 145 × 2 mm^3^. To cool the irradiation samples and the sample holders, a rectification lattice with square apertures is installed in front of the samples. The size of the rectification lattice is 52 × 52 × 5 mm^3^, and the size of each square aperture is 4 × 4 mm^2^. Furthermore, to cool the side wall of the sample holder, a 2 mm-wide slit is arranged, adjacent to each edge of the rectification lattice, to direct the LBE flow. When the LBE flow returns from the BW to pass through the rectification lattice area, its flow path shrinks sharply. Therefore, the rectification lattice acts as an orifice.

## 3. Heat Deposition in LBE

**Figure 2:**
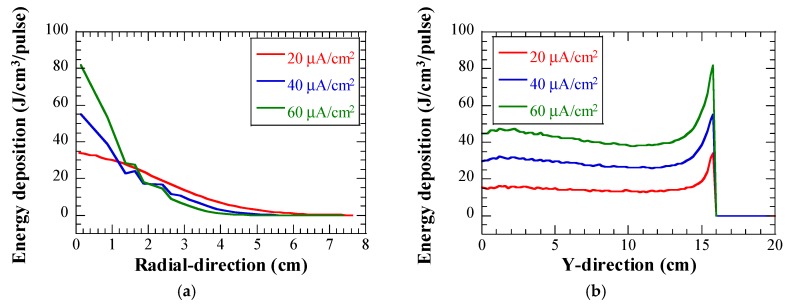
Energy deposition in LBE due to one pulse proton beam injection with various peak beam current densities: (**a**) In radial-direction, Y = 15.8 cm; and (**b**) in Y-direction, X = 0 cm.

Figure 2 shows heat deposition in the LBE, due to the injection of one pulse of proton beam with various peak beam current densities. Heat deposition data were obtained by using the Particle and Heavy Ion Transport code System (PHITS) code [8]. The reference proton beam conditions of LBE spallation target were used in the present study for the numerical calculations [9,10]. The proton beam had a Gaussian profile. The beam current of 625 μA will be the maximum beam current during the operation of the target test facility. The pulse duration of the proton beam was set to 0.5 ms. The maximum rates of energy deposition in the LBE (one pulse) were approximately 34 J/cm^3^, 55 J/cm^3^, and 82 J/cm^3^, for the beam current densities of 20 μA/cm^2^, 40 μA/cm^2^, and 60 μA/cm^2^, respectively. Considering that the Bragg-peak in the Y-direction appeared only 158 mm downstream of BW center, the length of the straight tube in the numerical simulation models was set to 300 mm, to ensure that the calculation of heat deposition in the LBE was not affected and to reduce the model size and calculation cost, to a great extent. It was expected that pressure waves would be generated in the LBE owing to the rapid depostion of large amounts of heat.

## 4. Numerical Simulation Models

### 4.1. Model for Calculating Dynamics of Pressure Waves

**Figure 3:**
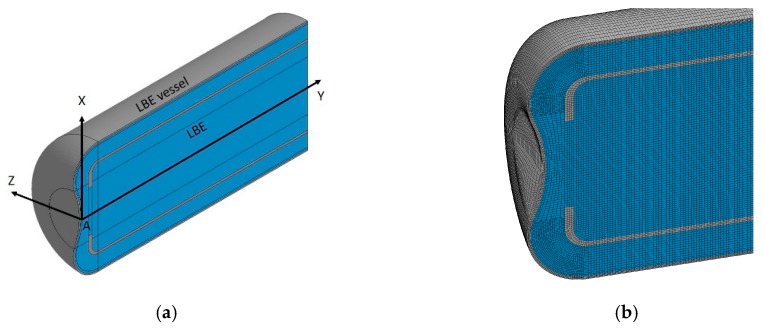
Schematic drawing of half 3D model of the LBE spallation target: (**a**) For calculating dynamics of pressure waves; and (**b**) an example of the mesh structure.

Figure 3a shows the analytical model for calculating pressure waves. Dynamic analyses were performed using LS-DYNA, a commercial Finite Element Method program [11]. The explicit code of LS-DYNA was used for calculation. The material properties used for calculating the pressure waves are listed in Table 1. A half three-dimensional (3D) model was prepared. The target vessel and the LBE were divided into hexahedral solid elements. The number of elements and nodes in the model were approximately 0.76 million and 0.83 million, respectively. The size of the first layer mesh that was the closest to the LBE vessel was ca. 0.5 mm, as can be seen in Figure 3b. Mesh sensitivity tests were performed, and this mesh structure was found to be capable of providing mesh-independent simulation results. The time step was 2.15 × 10^−8^ s, which was sufficiently small to ensure a stable explicit calculation. For the boundary condition, the maximum of the Y-direction was considered non-reflective. The solid liquid contact was set to an automatic contact type, according to which the sliding between the solid and the liquid was allowed, but not separation. Here the cut-off model was not applied to the boundary because the generated negative pressure was considerably small. For simplicity, the rectification lattice and irradiation samples were not included in the model. The rise in temperature of liquid metal due to the injection of proton beams, was assigned to the elements in the numerical simulation model, which could be calculated using Equation (1):

(1) $$\Delta T=\frac{Q}{\rho C_{P}},$$

where Q is the energy density in liquid metal (J/m^3^), ρ is the density of liquid metal (kg/m^3^), and $C_{P}$ is the specific heat of liquid metal (J/ kg·K).

Owing to a rise in temperature, the volume of heated liquid metal tended to increase. However, the surrounding liquid metal could not immediately be moved by this expansion; therefore, inverse constraints were imposed on the heated liquid metal and pressure was generated, which could be calculated using Equation (2):

(2) P=K·β·ΔT ,

where K is the bulk modulus (GPa), and β is the volume thermal expansion coefficient (1/K).

### 4.2. Computational Fluid Dynamics (CFD) Analysis Model

**Figure 4:**
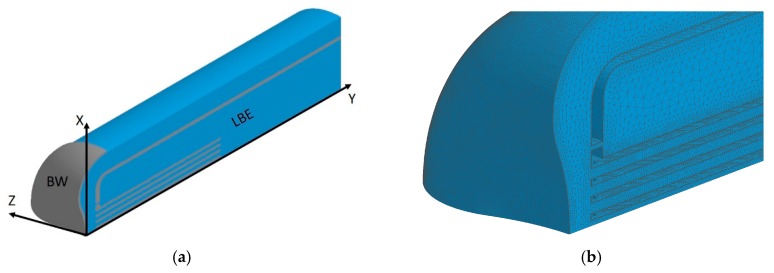
Schematic drawing of a quarter 3D model of the LBE spallation target: (**a**) For CFD analysis; and (**b**) an example of the mesh structure of LBE.

To study the turbulent LBE flow, we conducted steady-state CFD analyses using STAR-CD (Version 4.26, Siemens Product lifecycle Management Inc., Plano, TX, USA), a commercially available code. In the analysis model, the BW part of the target was built but not in the LBE vessel part, as shown in Figure 4a. The Reynolds number, Re, was larger than 3 × 10^4^ under nominal operation condition of the LBE spallation target; hence, turbulent flow was formed in the flow channel. The high-*Re k*-epsilon (*k-ε*) turbulence model was adopted, which was considered to be suitable to simulate the main LBE flow features in the flow channel.

The number of tetrahedral meshes in the quarter model was approximately 2.8 million, which meant that the mesh size was sufficiently fine to obtain an adequate spatial resolution for simulating the LBE flow. Five layers of prism meshes were extruded on the LBE surface, to connect it to the LBE vessel. The distance of the first node closest to the wall in the boundary layer was 0.1 mm, as shown in Figure 4b. Therefore, the y+ value was less than 100, which was suitable for the *k-ε* turbulence model. In the transient analyses, the time step was 10^−5^ s, so the Courant number was far less than 1, which guaranteed solution convergence. Mesh sensitivity tests were performed as well, and mesh-independent results were obtained, using the present mesh structures. The effects of gravity on LBE flow was considered in the simulations. A non-slip condition was applied at the wall surface with zero velocity. 

At the beginning, we focused on the nominal operation condition of the LBE spallation target in the CFD analysis. The inlet flow rate in the annular channel was set to 1 × 10^−6^ m^3^/s, at a temperature of 350 °C. The inlet velocity around the annular channel was assumed to be uniform. LBE at this flow rate was adequate to cool the BW, while avoiding severe erosion/corrosion damage to the target vessel, owing to the rapid LBE flow. The pressure at the outlet boundary was set to a constant value of zero. The outer surface condition of the BW was set to adiabatic, because a very small amount of heat is lost by conduction and radiation from the BW to the surrounding vacuum environment.

## 5. Results and Discussions

### 5.1. Cavitation Damage due to Pressure Waves

#### 5.1.1. Time Response of Pressure

**Figure 5:**
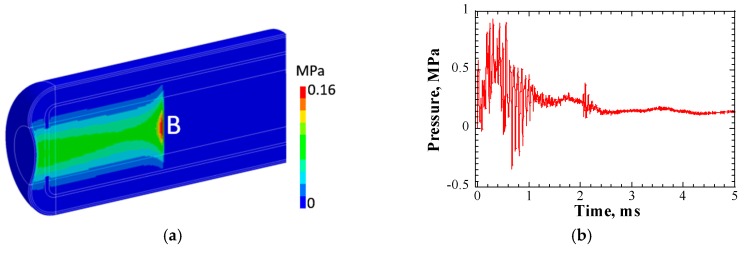
(**a**) Pressure distribution contour (at 1 μs); and (**b**) time response of pressure at position of maximum heat deposition position in LBE (position B). Beam density = 20 μA/cm^2^.

Figure 5 shows the pressure distribution contour and the time response of pressure at the position of maximum heat deposition position, in the LBE spallation target. The beam density was 20 μA/cm^2^. The pressure distribution contour was obtained at 1 μs. After injection of the proton beam, positive pressure in the LBE target rose immediately. On the one hand, the pressure at maximum heat deposition increased as the temperature of the LBE increased. On the other hand, pressure waves were generated, owing to rapid expansion and spreading around of LBE, while the propagation of the pressure waves led to a decrease in pressure at the position of maximum heat deposition. Owing to the effects of the two above-mentioned factors, the pressure at the maximum heat deposition position increased to 0.6 MPa at 13 μs and then decreased subsequently. As the pulse duration was 0.5 ms, the pressure wave dynamics became very complex and pressure waves propagating from various directions or those reflected by the boundaries were superimposed on each other, at the detection position.

**Figure 6:**
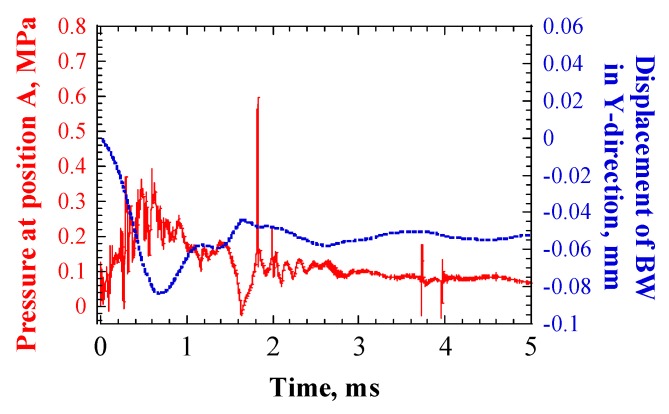
Example of time response of pressure at position A (in Figure 3) and displacement of the Beam Window (BW) in Y-direction; beam density = 20 μA/cm^2^.

Figure 6 shows an example of the time response of pressure at Position A, which is marked in Figure 3, and displacement of the BW in Y-direction. Position A was where the LBE contacted the target vessel at the center of the BW. The peak beam current density was 20 μA/cm^2^. The high-frequency components in the time responses could be attributed to an impulsive contact between the LBE and target vessel, during pressure wave propagation. The maximum negative pressure peak of ca. −0.03 MPa appeared at 1.6 ms, followed by a positive pressure of ca. 0.6 MPa at 1.8 ms.

**Figure 7:**
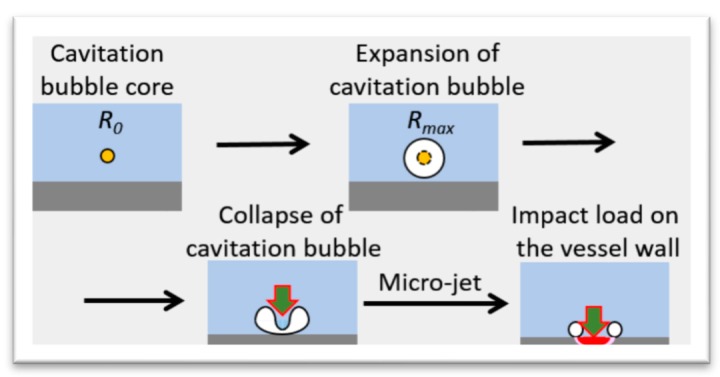
Schematic drawing of the mechanism of cavitation damage occurrence.

The pressure waves shocked the target vessel, which deformed the center of the BW along the negative Y-direction. This sudden deformation of the vessel subsequently pulled the adjacent LBE, so the volume of LBE increased, which induced the generation of negative pressure in the LBE [12]. The core of the cavitation bubble in the LBE grew in the upward direction, owing to the negative pressure. After reaching its maximum size, at which point the forces acting on the bubble were in equilibrium, it collapsed. The collapse of the cavitation bubble released a shock wave on the target vessel, which caused cavitation damage. A schematic drawing of the mechanism of cavitation damage is shown in Figure 7.

#### 5.1.2. Threshold of Cavitation Damage Initialization

It is known that the development of cavitation damage can be divided into two stages: incubation and steady-state period. The former stage is the initial stage of cavitation damage, in which the material surface undergoes a plastic deformation, owing to the intense pressure released by the collapse of cavitation bubbles. With the accumulation of cavitation damage, cracks are formed, and the material surface is peeled off from the bulk, which is indicated by a significant mass loss in the latter stage.

The initiation of cavitation damage on the material surface is closely related to the magnitude of pressure released by the collapse of cavitation bubbles. The pressure released can be determined based on the expansion ratio of the cavitation bubbles, which is the ratio of maximum bubble radius ($R_{max}$) to radius of bubble core ($R_{0}$). The bubble core is the initial nuclei of cavitation bubbles. It has been found that the incubation period of cavitation damage is proportional to the third power law of the cavitation bubble expansion ratio [13]. A parameter called “cavitation intensity” (D) was proposed. Its correlation with the cavitation bubble expansion ratio can be quantitatively expressed using [14]:

(3) $$D=f\left({\left(\frac{R_{max}}{R_{0}}\right)}^{3}\right)\;,$$

The growth behavior of cavitation bubbles depends on the amplitude and time duration of negative pressure. The growth dynamics of a single bubble in a pressure wave field can be calculated using the Keller equation [15,16]:

(4) $$\left(1-\frac{\dot{R}}{C}\right)R\ddot{R}+\left(\frac{3}{2}-\frac{\dot{R}}{2C}\right){\dot{R}}^{2}=\frac{1}{\rho }\left(1+\frac{\dot{R}}{C}\right)\left(P_{b}\left[t\right]-P\left[t+\frac{R}{C}\right]-P_{0}\right)+\frac{R}{\rho C}{\dot{P}}_{b}\left[t\right]\;,$$

(5) $$P_{b}\left[t\right]=P_{g}\left[t\right]-\frac{2\sigma +4\eta \dot{R}}{R},$$

(6) $$P_{g}\left[t\right]=\left(P_{0}-P_{V}+\frac{2\sigma }{R}\right){\left(\frac{R_{0}}{R}\right)}^{\gamma }+P_{V},$$

where R is the time-dependent bubble radius (mm), $R_{0}$ is radius of the bubble core (mm), C is the velocity of sound in liquid (m/s), ρ is density of liquid metal (kg/m^3^), σ is surface tension (N/m), η is viscosity of liquid metal (Pa·s), γ is specific heat ratio, $P_{g}$ is pressure in the bubble (Pa), $P_{b}$ is pressure in the liquid surrounding the bubble (Pa), $P_{V}$ is vapor pressure (Pa), $P_{0}$ is static pressure in the liquid (Pa), and P is pressure oscillation near the bubble (Pa).

In Japan Atomic Energy Agency (JAEA), cavitation damage to the mercury spallation target of the J-PARC, due to pressure waves, has been studied systematically by using an electro-Magnetic Impact Testing Machine (MIMTM). Specimens made of type 316 SS, without any surface treatment, were used in those tests. It was noticed that the incubation period of the cavitation damage extended to approximately 10^9^ pulses of proton beam injections, and there was hardly any cavitation damage on the test specimens, when the MIMTM power was lower than 185 W [17,18,19]. In this case, the maximum cavitation bubble radius was approximately 250 μm, as given in Reference [20].

It is known that the equilibrium radius, $R_{EQ}$, of a spherical bubble filled with gas or vapor, can be expressed as follows [21]:

(7) $$P_{V}-P_{\infty }-P+P_{g}-\frac{2\sigma }{R_{EQ}}=0,$$

(8) $$P_{g}=P_{g0}{\left(\frac{R_{0}}{R_{EQ}}\right)}^{3\kappa },$$

where $P_{\infty }$ is the static pressure of the liquid far from the bubble (Pa), $P_{g}$ is partial pressure of the gas in the bubble (Pa), $P_{g0}$ is initial partial gas pressure in the bubble (Pa) (can be calculated from Equation (6)), and κ is polytropic constant. It should be noted that pressure oscillation, P, is considered in Equation (7). For a given mass of gas in a bubble, $m_{G}>0$ (which means there is gas in the bubble in addition to vapor), and if one plots ($P_{V}-P_{\infty }$) as a function of $R_{EQ}$, there would be a turning point on the equilibrium radius curve; this equilibrium radius is called the Blake’s threshold radius. Here, it is called the critical radius, $R_{C}$, and it was calculated as follows [21,22]:

(9) $$R_{C}={\left(\frac{9\kappa m_{G}T_{B}K_{G}}{8\pi \sigma }\right)}^{1/2},$$

(10) $$m_{G}=\frac{4P_{g0}R_{0}{}^{3}\pi }{3T_{\infty }K_{G}},$$

where $m_{G}$ is the mass of gas in the bubble, $K_{G}$ is the gas constant, $T_{B}$ is bubble temperature (K), and $T_{\infty }$ is ambient temperature (K). If the bubble radius $R_{EQ}<R_{C}$, the bubble is said to be in a stable equilibrium status, even under pressure oscillation; by contrast, bubbles with radius $R_{EQ}>R_{C}$ are unstable, which means they grow upward or collapse. $R_{C}$. depends on the amplitude of the generated pressure oscillation. Here, only the maximum negative pressure point was considered, during the time response of the pressure curve, because the amplitude of the maximum pressure is a factor that determines the size of a cavitation bubble after its growth.

In the case of MIMTM experiments, the maximum negative pressure was approximately −0.11 MPa, when the MIMTM power was 185 W [20]. According to Equations (7–10), the value of $R_{C}$ was calculated as approximately 8 μm, so the expansion ratio of a cavitation bubble was approximately 31.5. That is, the threshold of the expansion ratio of a cavitation bubble that can initialize cavitation damage was approximately 31.5. Considering that the physical properties related to bubble dynamics are similar between mercury and LBE, and cavitation intensity is mainly determined by the expansion ratio rather than the absolute bubble size, it is reasonable to assume that this threshold is suitable for the LBE target, as well. In case of the LBE target, the maximum negative pressure was –0.03 MPa, under the beam conditions shown in Figure 6; accordingly, $R_{C}$ equaled approximately 22 μm, which meant only bubbles of a radius larger than 22 μm, could grow upward.

#### 5.1.3. Cavitation Bubble Expansion for LBE Spallation Target

**Figure 8:**
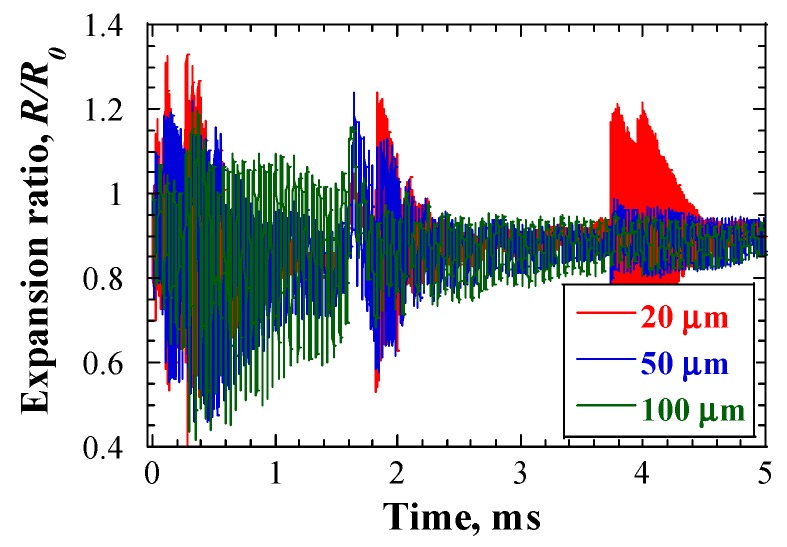
Time response of the expansion ratio of cavitation bubbles at Position A, under initial bubble core radii conditions; beam current density = 20 μA/cm^2^.

**Figure 9:**
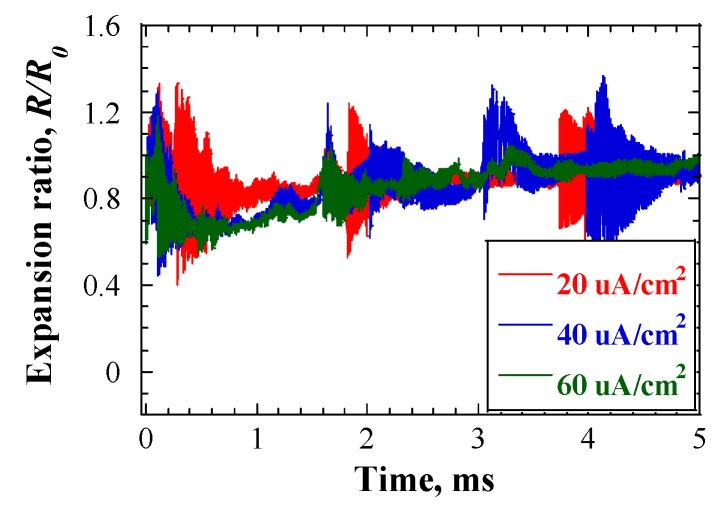
Time response of expansion ratio of cavitation bubbles at Position A, under various proton beam conditions; *R_0_* = 20 μm.

Figure 8 shows the time responses of cavitation bubble dynamics, as calculated using Equations (4–6), in cases of various initial cavitation bubble radii. The beam current density was 20 μA/cm^2^. The responses are shown in the form of bubble expansion ratio, $R/R_{0}$. The bubble expansion ratio was smaller than 1.4 in all cases. The effects of beam current density on the bubble expansion ratio were studied as well, and the results are shown in Figure 9. The bubble core radius, $R_{0}$, was set to 20 μm. Although the beam current density increased from 20 μA/cm^2^ to 60 μA/cm^2^, the maximum bubble expansion ratio did not exceed 1.4. This bubble expansion ratio was considerably lower than the threshold, which was discussed in the previous sub-section. Such a small expansion ratio of cavitation bubbles would not cause severe cavitation damage on the LBE vessel.

In case of the J-PARC mercury target, the expansion ratio of cavitation bubbles could be more than 100 when the proton beam power was 1 MW. Compared to the J-PARC mercury target, although the temperature rise in HLM was much higher in case of the LBE spallation target, the considerably smaller expansion ratio of cavitation bubbles was attributed to a significantly lower rate of pressure increase. For the mercury target, heat deposition due to one proton beam pulse in mercury was 17.2 J/cm^3^ and the resulting temperature rise was 9.2 K; therefore, the maximum pressure generated was 43 MPa, according to Equations (1–2). Pulse duration of the proton beam was 1 μs, in the case of the mercury target, so the rate of pressure rise was 43 MPa/μs. By contrast, in the case of the LBE spallation target, when the heat deposition in the LBE due to one proton beam pulse was 34 J/cm^3^ (20 μA/cm^2^), the maximum rise in the temperature of the LBE was 22.4 K, and the maximum pressure generated was 89.5 MPa. However, the pulse duration of the proton beam, in case of the LBE spallation target was 500 μs, so that the pressure rising rate was 0.18 MPa/μs. Therefore, the intensity of the pressure waves generated in the LBE was considerably weaker than that in mercury, leading to a considerably weaker and shorter duration of the negative pressure generated in the LBE adjacent to the BW. The resulting cavitation bubble expansion ratio was considerably smaller in the LBE.

### 5.2. Cavitation Damage due to LBE Flow

#### 5.2.1. Steady-State Flow

**Figure 10:**
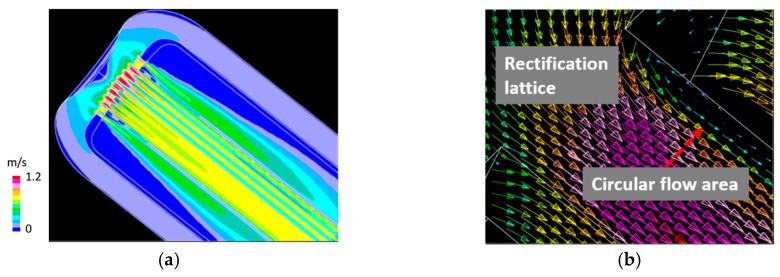
(**a**) LBE flow velocity contour of the spallation target head; and (**b**) an example of LBE flow velocity vector in rectification lattice area; inlet flow speed = 0.125 m/s.

The LBE flow velocity contour of the spallation target head is shown in Figure 10a. The nominal inlet LBE flow speed was 0.125 m/s. The maximum flow speed of the LBE was in the rectification lattice region, and its magnitude was 1.2 m/s. An example of the LBE flow velocity vector in the rectification lattice area is shown in Figure 10b. As anticipated, a circular flow region was formed downstream of the entrance of the rectification lattice area.

**Figure 11:**
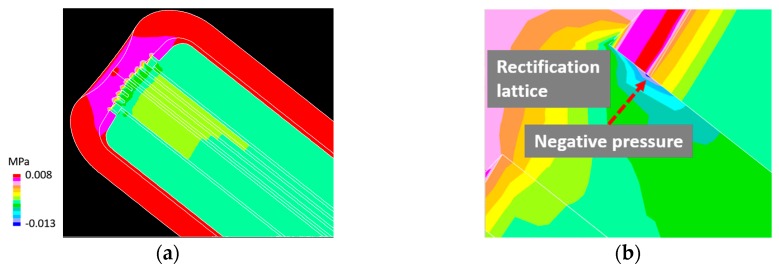
(**a**) Pressure distribution contour in LBE for the spallation target head; and (**b**) an example of pressure distribution contour in the LBE for the rectification lattice area; inlet flow speed = 0.125 m/s.

Figure 11 shows the pressure distribution contour in the LBE for the LBE spallation target head and an example of the pressure distribution contour in the LBE for the rectification lattice area. A maximum negative pressure of approximately −0.013 MPa was generated in the circular LBE flow region downstream of the rectification lattice entrance, which could be attributed to the pressure loss owing to the formation of the flow vortex.

**Figure 12:**
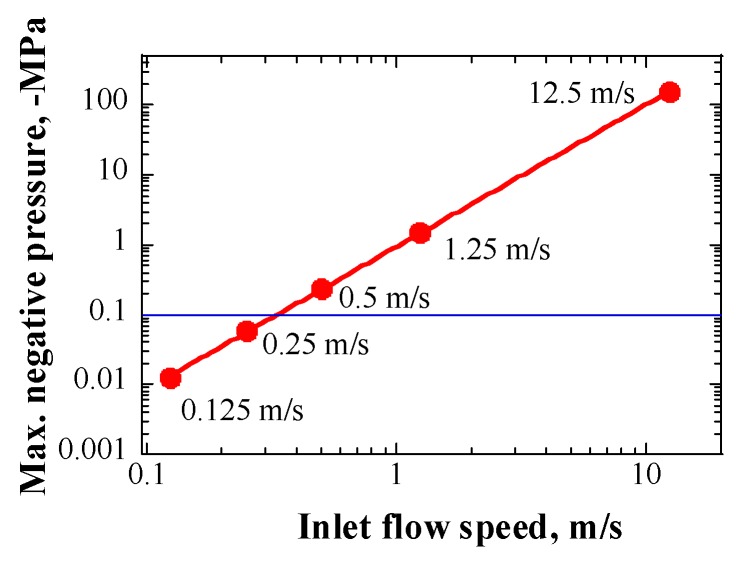
Maximum negative pressure generated in LBE as a function of LBE inlet flow speed.

Figure 12 shows the maximum negative pressure generated in the LBE, as a function of the LBE inlet flow speed. The maximum magnitude of the negative pressure in the LBE had a second power law relationship with the LBE inlet flow speed. The growth of a cavitation bubble core under hydrostatic conditions depends on many factors, such as gas pressure in the core, size of core, surface tension, and saturated vapor pressure in liquid. In general, for a looped system, to drive the growth of cavitation bubble cores, the sum of magnitudes of the negative pressure in the liquid and the saturated vapor pressure of the liquid should be larger than the external pressure at least, that is, the sum of the cover gas pressure and the surface tension. Here, only the cover gas pressure, which was 0.1 MPa in the case of the LBE target system, was considered, because the magnitude of the surface tension was negligible. The saturated vapor pressure of the LBE had the following relationship with temperature [23]:

(11) $$P_{s\left(LBE\right)}\left[Pa\right]=1.22\times 10^{10}\cdot exp\left(-\frac{22552}{T}\right)K$$

According to Equation (11), the saturated vapor pressure of LBE at around 600 K was only approximately 5.77 × 10^−7^ Pa, and it could be neglected. Therefore, to drive the growth of cavitation bubble cores, the inlet LBE flow speed should have been greater than 0.4 m/s, to generate a negative pressure adequate for overcoming the cover gas pressure, as can be deduced from the relationship shown in Figure 12. However, during normal operations of the LBE spallation target, LBE flow speed did not hit 0.4 m/s, as the maximum inlet flow speed was 0.25 m/s, considering the ability of the electro-magnetic pump (EMP).

In addition, the cavitation number, $C_{a}$, was considered in the rectification lattice region, the definition of which is as follows:

(12) $$C_{a}=\frac{P_{d}-P_{V}}{\frac{1}{2}\rho V_{L}{}^{2}},$$

where $P_{d}$ is the pressure, downstream of the rectification lattice region (Pa), and $V_{L}$ is the flow speed of the liquid (m/s). Under the reference flow condition, $C_{a}$ is less than 0.1. It has been reported that the incipient cavitation number in PbBi-68 (chemical composition, mass%: 50% Bi, 26.7% Pb, 13.3% Sn, and 10% Cd; melting point: 68 °C) that flows through an orifice, should reach approximately 0.7, when the $R_{e}$ (Reynolds number) ranged from (5.8−7.4) × 10^4^ [24]. The $R_{e}$ in the rectification lattice region of the LBE target had a similar value. Therefore, based on the value of the cavitation number, cavitation would not occur in the rectification lattice. To summarize, it was considered that the cavitation damage would likely not be caused by the turbulent flow of LBE, under a normal steady-state flow condition.

#### 5.2.2. Transient Flow

**Figure 13:**
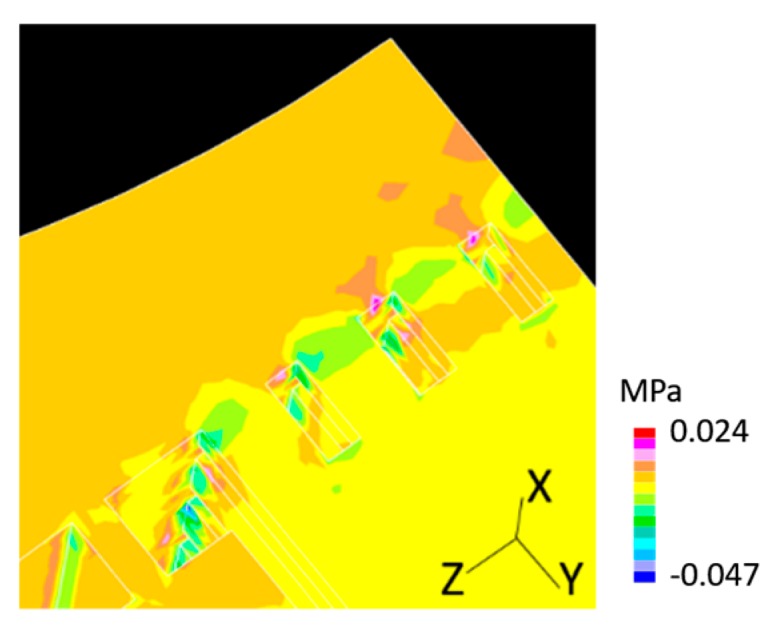
Pressure distribution contour in LBE for the transient analysis at 10 μs.

During operation of the LBE spallation target, the LBE flow rate might decrease suddenly, owing to the malfunction of the EMP. In this case, the LBE flow speed would decrease from the nominal 0.125 m/s to 0 m/s, in approximately 100 s, and thus, the rate of change of inlet flow speed was 0.00125 m/s^2^, according to the test performed using a mock-up loop of the target primary cooling system. Transient analysis was performed under this transient flow condition, as the first step. Figure 13 shows a snapshot of the averaged pressure distribution contour in the LBE; the time-point was 10 μs. Negative pressure with a maximum magnitude of approximately 0.047 MPa, was generated in the rectification lattice region, owing to the pressure change caused by a decrease of flow in velocity.

**Figure 14:**
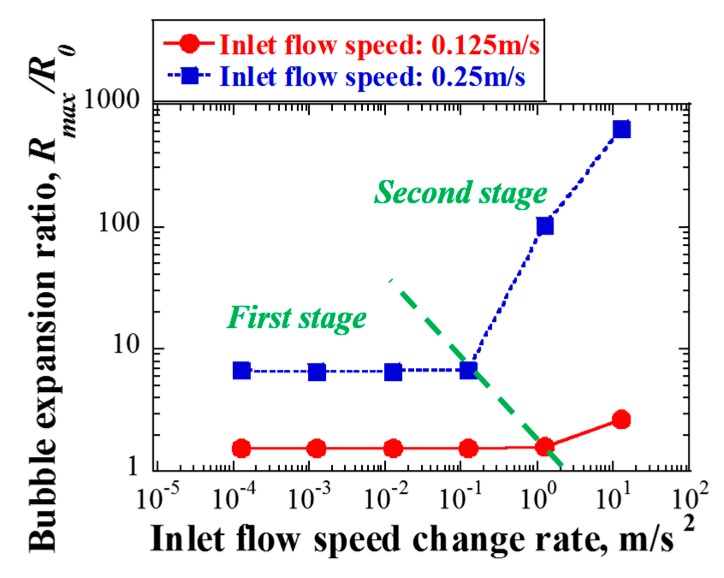
Bubble expansion ratio as a function of rate of change in inlet flow speed for various inlet flow speeds.

The bubble expansion ratio was plotted in Figure 14, as a function of the inlet flow speed change rate for various initial inlet flow speeds. In addition to the nominal flow speed, the maximum inlet flow speed (0.25 m/s) was considered. In fact, given an inlet flow speed of 0.125 m/s, the expansion ratio was only approximately 2.7, even when the rate of change in flow speed increased to 12.5 m/s^2^. However, when the initial inlet flow speed was 0.25 m/s, the expansion ratio increased abruptly to more than 100 and 600, when the rates of change in flow speed were 1.25 m/s^2^ and 12.5 m/s^2^, respectively. Such a large expansion ratio was adequate for causing severe cavitation damage on the vessel wall. The above results suggest that cavitation damage would perhaps not have occurred under the nominal flow condition, even if the inlet flow speed decreased from 0.125 m/s to 0 m/s, in 0.01 s, which was apparently an assumed extreme abnormal state of target operation. However, in the case of 0.25 m/s, if the rate of change in inlet flow speed exceeded approximately 0.2 m/s^2^, the expansion ratio of the cavitation bubbles would be greater than 12.5 as a result; in such a case, the possibly of a cavitation damage could not have been avoided.

Furthermore, it can be deduced from Figure 14 that the variation of maximum bubble expansion ratio, $E_{m}$, can be divided into two stages. In the first stage, $E_{m}$ is related only to the inlet flow speed, while seeming independent of the rate of change in inlet flow speed, and the relationship can be expressed as follows:

(13) $$log\left(E_{m}\right)=a\cdot log\left(V_{i}{}^{2}\right),$$

where $V_{i}$ is the inlet flow speed (m/s), and a is a constant related to the material property.

In the second stage, $E_{m}$ is related to both the inlet flow speed and the rate of change in inlet flow speed, and the relationship can be expressed as follows:

(14) $$log\left(E_{m}\right)=b\cdot log\left(V_{i}{}^{c}\cdot {\dot{V_{l}}}^{d}\right),$$

where ${\dot{V}}_{l}$ is the inlet flow speed change rate (m/s^2^), and b, c, and d are constants related to the material property. 

**Figure 15:**
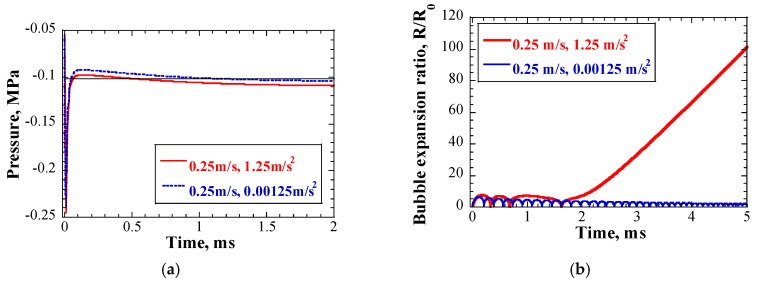
(**a**) Time response of pressure at position of maximum negative pressure; and (**b**) time response of single bubble dynamics; inlet flow speed = 0.25 m/s.

Figure 15a shows an example of the time response of pressure and time response of single bubble dynamics. The initial inlet flow speed was 0.25 m/s, and the rates of change in the inlet flow speed were 1.25 m/s^2^ and 0.00125 m/s^2^, respectively. The solid black line represent the initial pressure in the bubble. The negative pressure in the case of 1.25 m/s^2^ was lower than that in the case of 0.00125 m/s^2^, and it was sustained for a longer time, under the bubble initial pressure line, which resulted in a considerably higher bubble expansion ratio, relative to that in the case of 0.00125 m/s^2^, as shown in Figure 15b. The amplitude of negative pressure and its duration were dominant factors governing the growth behavior of a cavitation bubble.

## 6. Conclusions

In this study, cavitation damage to the LBE spallation target, due to pressure change in HLM, was studied through numerical simulations from the viewpoint of single bubble dynamics; the results are summarized below:

1. The intensity of pressure waves generated in the LBE was found to be weak due to the relatively long duration of the proton beam pulse. Therefore, the expansion ratio of the cavitation bubbles due to the pressure waves, was only 1.4, which was considerably lower than the threshold ratio that could lead to severe cavitation damage on the vessel.
2. The magnitude of maximum negative pressure had a second power law relationship with the flow speed of the HLM. For the nominal inlet flow speed of 0.125 m/s, the negative pressure induced by the steady-state LBE flow was only −0.013 MPa, which was considerably smaller than the cover gas pressure of the LBE spallation target; therefore, this pressure could not drive the growth of the cavitation bubbles.
3. For the transient LBE flow, negative pressure was generated in the LBE, due to a decrease in LBE flow velocity. Under normal target operation conditions, the duration of negative pressure was too short to drive the growth of adequately large cavitation bubbles. However, cavitation might have occurred under a few extreme flow variation conditions, for example, when the rate of change in inlet flow was higher than 0.2 m/s^2^, even as the initial inlet flow speed was 0.25 m/s.
4. The maximum cavitation bubble dynamics due to turbulent flow in an orifice could be classified into two stages. In the first stage, the maximum cavitation bubble expansion ratio shared a power law relationship with the inlet flow speed, but it was almost independent of the inlet flow speed change rate; in the second stage, the maximum cavitation bubble expansion ratio shared, a power law relationship with, both, the inlet flow speed and the rate of change in the inlet flow speed.

It should be mentioned that all results reported, herein, were those of numerical simulations. In the future, a loop experiment will be performed to study the cavitation damage issue by investigating the flow dynamics of the LBE spallation target.

## Figures and Tables

**Table 1:** Physical properties of 316 SS and LBE for calculation of pressure waves.

Physical Properties	Symbol	Unit	316 SS	LBE
Density	ρ	kg/m^3^	7908	10450
Young’s modulus	E	MPa	1.742 × 10^5^	92.8
Poisson’s ratio	ν	-	0.3153	0.4995
Thermal expansion coefficient	β	K^−^^1^	-	1.285 × 10^−4^
